# PET: Parameter-efficient Knowledge Distillation on Transformer

**DOI:** 10.1371/journal.pone.0288060

**Published:** 2023-07-06

**Authors:** Hyojin Jeon, Seungcheol Park, Jin-Gee Kim, U. Kang

**Affiliations:** Department of Computer Science and Engineering, Seoul National University, Seoul, Republic of Korea; Huazhong University of Science and Technology, CHINA

## Abstract

Given a large Transformer model, how can we obtain a small and computationally efficient model which maintains the performance of the original model? Transformer has shown significant performance improvements for many NLP tasks in recent years. However, their large size, expensive computational cost, and long inference time make it challenging to deploy them to resource-constrained devices. Existing Transformer compression methods mainly focus on reducing the size of the encoder ignoring the fact that the decoder takes the major portion of the long inference time. In this paper, we propose PET (Parameter-Efficient knowledge distillation on Transformer), an efficient Transformer compression method that reduces the size of both the encoder and decoder. In PET, we identify and exploit pairs of parameter groups for efficient weight sharing, and employ a warm-up process using a simplified task to increase the gain through Knowledge Distillation. Extensive experiments on five real-world datasets show that PET outperforms existing methods in machine translation tasks. Specifically, on the IWSLT’14 EN→DE task, PET reduces the memory usage by 81.20% and accelerates the inference speed by 45.15% compared to the uncompressed model, with a minor decrease in BLEU score of 0.27.

## Introduction

*How can we compress a large Transformer model into a smaller model which maintains the original performance?* Transformer [1] has achieved state-of-the-art performance in the field of Natural Language Processing (NLP) [2–4]. It has shown its potential in a variety of practical applications such as language modeling, translation, and question-and-answering. These applications run in various environments including mobile devices. However, most mobile devices have restricted memory size and poor computation abilities. Also, low energy consumption and fast inference speed are important in the real world. On the contrary, enlarging a language model and improving its performance has been the main trend in NLP for the last few years. More and more large models have been introduced and achieved remarkable performance. Latest models, such as GPT-3 [5] and Megatron-Turing NLG [6] have more than hundreds of billions of parameters. However, their excessive memory usage, energy consumption, and long inference time prohibit them from being deployed to resource-limited devices or real-world applications despite their outstanding competence [7, 8]. Therefore, it is crucial to compress Transformer models efficiently.

Recently, several Transformer compression methods have been proposed [9–13]. However, most studies mainly attend to compressing the encoder, including the works on BERT [2] compression (e.g., DistilBERT [14], TinyBERT [15], MiniLM [16, 17], DynaBERT [18], MobileBERT [7], Pea-KD [19] and SensiMix [20]).

Many NLP tasks such as translation and speech recognition are dependent on both the encoder and the decoder of the Transformer model. The decoder accounts for half of the entire model size and is the main cause of the long inference time of Transformer models. It is not easy to directly apply existing BERT compression methods when compressing the decoder since the encoder and the decoder have different architectures. Therefore, we need an efficient and flexible method for Transformer compression which shrinks the size of both the encoder and the decoder.

In this paper, we propose PET (Parameter-Efficient knowledge distillation on Transformer), an accurate Transformer compression method that reduces the model size, computational cost, and inference time while conserving the accuracy of the original model. PET compresses the size of both the encoder and decoder of the Transformer simultaneously. PET exploits Knowledge Distillation on Transformer while optimizing the structure of student model and the process of initialization. PET utilizes different sharing patterns for the encoder and decoder considering their characteristics. Furthermore, PET succeeds in pre-training a student model more efficiently and enhances the model’s accuracy.

Our main contributions are as follows:

- **Algorithm**. We propose PET, an efficient Transformer compression method that reduces the size of both the encoder and decoder of the Transformer while minimizing performance degradation.
- **Generality**. The techniques introduced by PET can be applied to other types of Transformer-based language models.
- **Performance**. Extensive experiments on multiple real-world language datasets show that PET consistently achieves better performance than competitors. PET achieves memory, computation, and inference time improvements by up to 81.20%, 80.16%, and 45.20%, respectively, with BLEU score loss below 1.

In the rest of the paper, we first explain preliminaries about the Transformer model and review existing Transformer compression methods based on Knowledge Distillation. Then, we describe the proposed algorithm PET in detail and present experimental results to evaluate PET. We summarized the symbols frequently used in this paper in Table 1. The code of PET is available at https://github.com/snudm-starlab/PET.

**Table 1:** Table of symbols.

Symbol	Description
*L*_*i*_	*i*-th layer
*f*_*t*_, *f*_*s*_	teacher and student model
$Q_{i}^{\text{self}}$ , $K_{i}^{\text{self}}$,$V_{i}^{\text{self}}$	query, key, and value matrices of the self-attention sub-layer in the *i*-th layer
$Q_{i}^{\text{ed}}$ , $K_{i}^{\text{ed}}$, $V_{i}^{\text{ed}}$	query, key, and value matrices of the encoder-decoder-attention sub-layer in the *i*-th layer

## Related work

### Transformer

Transformer [1] was introduced for sequence-to-sequence tasks such as summarization and translation. It maps a source sequence to a target sequence, e.g., translating English to German.

#### Architecture

The Transformer model uses an encoder-decoder architecture. The encoder encodes the input sequences from the source domain and feeds the code to the decoder. Then, the decoder generates token-wise outputs in an auto-regressive way using the received code and its previous outputs. The encoder and the decoder have multiple layers consisting of two types of sub-layers: a multi-head attention mechanism and a position-wise fully connected feed-forward network. There are three types of multi-head attention sub-layers in the Transformer model. The first one is a multi-head self-attention which is used in the encoder. Multi-head self-attention receives the output of the previous encoder layer and utilizes it for the query, key, and value. Masked multi-head self-attention in the decoder is similar to multi-head self-attention; however, it attends only to the previous tokens excluding the subsequent ones. Encoder-decoder multi-head attention is used in the decoder and it captures the relationship between the encoded code and the output of the masked multi-head self-attention in the previous sub-layer. Encoder-decoder multi-head attention uses the output of the previous sub-layer as the query and the output of the encoder as the key and value.

#### Output structure

**Fig 1:**
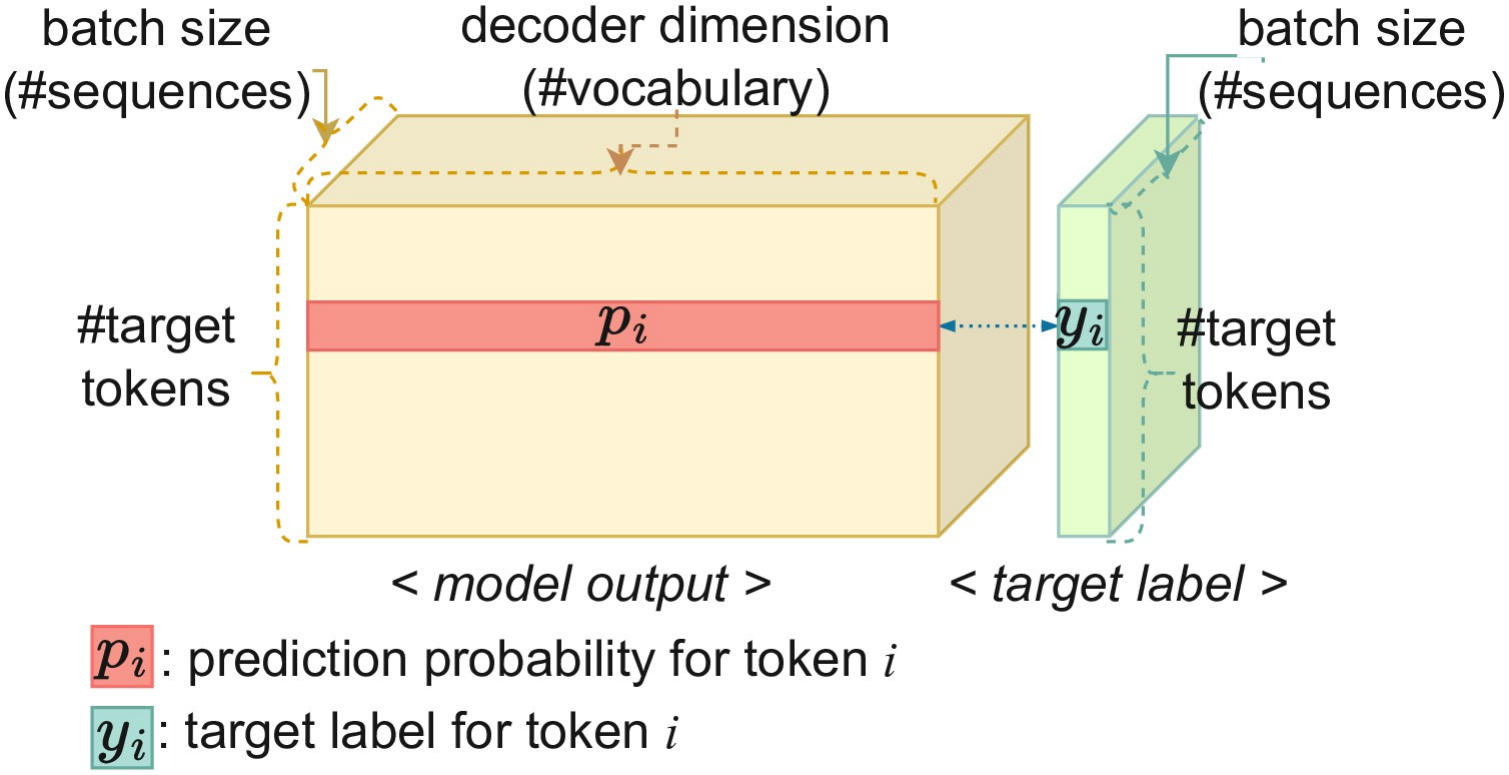
The model output structure of Transformer. The final output of the Transformer is a tensor of three dimensions. The three dimensions represent the number of output sequences in a batch, the number of tokens in each sentence, and the number of the target vocabulary, respectively. *p*_*i*_ is the prediction probability for token *i*. *y*_*i*_ is the target label for the token *i*.

The output structure of the Transformer is illustrated in Fig 1. The final output of the Transformer is a three-dimensional tensor. The three dimensions represent the number of output sequences in a batch, the number of tokens in each sentence, and the number of the target vocabulary, respectively. The model predicts the probability for each token over the entire target vocabulary. In Fig 1, *p*_*i*_ is the prediction probability of the token *i*. The vocabulary of the largest probability is selected as the predicted answer to the token *i* and compared with the target label *y*_*i*_.

#### Multi-head attention

The multi-head attention uses multiple attention heads with different projection layers to capture diverse patterns of attention maps. A detailed computation of the multi-head attention for query *Q*, key *K*, and value *V* is as follows:

$$\begin{array}{c} \begin{array}{cc} \text{MultiHead}(Q,K,V) & =\text{concat}({\text{head}}_{1},\ldots ,{\text{head}}_{n_{h}})\\ {\text{head}}_{i} & =\text{Attention}(QW_{i}^{Q},KW_{i}^{K},VW_{i}^{V})\\ \text{Attention}(QW_{i}^{Q},KW_{i}^{K},VW_{i}^{V}) & =\text{softmax}(\frac{\left(QW_{i}^{Q}\right){\left(KW_{i}^{K}\right)}^{T}}{\sqrt{d_{k}}})\left(VW_{i}^{V}\right) \end{array} \end{array}$$ (1)

where *n*_*h*_ is the number of the attention heads and *d*_*k*_ is a dimension of the key. $W_{i}^{Q}$, $W_{i}^{K}$, $W_{i}^{V}$ are projection matrices.

### Knowledge Distillation on Transformer

Knowledge Distillation (KD) [21] transfers knowledge of a larger, complex model (teacher) to a smaller one (student) to obtain a compact model that retains the accuracy of the larger one. It is a widely known and efficient approach for compressing various models such as Convolution Neural Networks (e.g., FALCON [22], Aghli et al. [23]) and Graph Convolution Networks (e.g., MustaD [24], MSKD [25]). Unlike unstructured pruning [26] and quantization [20, 27], KD does not require any specialized hardware or libraries to speed up the compressed models [28]. Also, it is compatible with other state-of-the-art works and easily takes advantage of them [18].

The overall process of KD is as follows: Given a large and well-trained teacher model, we first construct a small student model and initialize it. Then, we train the student model using the prediction outputs of the teacher model in addition to the ground truth labels for the target. Recent KD-based approaches [7, 14–19, 29–32] further utilize intermediate features of the teacher model with different loss function designs, allowing the student model to capture more knowledge of the teacher. We classify Transformer compression methods based on KD according to their compression targets: whether they compress only the encoder, only the decoder, or both. We focus on how each method constructs and initializes the smaller student model.

#### Knowledge Distillation on Transformer encoders

KD on BERT falls into this category. Patient-KD [29] extracts knowledge from intermediate layers as well as the final prediction. It initializes the student model by taking several layers of the teacher. DistilBERT [14] introduces a triple loss combining task, distillation, and cosine-distance losses. It initializes the student model by taking one of the two layers of the teacher. However, Patient-KD and DistilBERT constrain the architecture of the student model so that the dimensions of layers align with those of the teacher model. This limits the potential for achieving further compression. TinyBERT [15] distills attention matrices and hidden states of the Transformer with two-stage learning: general and task-specific distillation. It initializes the student model for task-specific distillation with another model trained at task-general distillation. MobileBERT [7] requires a specially designed teacher model equipped with an inverted-bottleneck structure to distill the knowledge to train the student model. TinyBERT and MobileBERT succeed in obtaining a more compact model than previous works with smaller dimensions for layers. However, they still have a limitation in that they introduce extra parameters or a specially designed intermediate model (i.e., IB-BERT) to address the mismatch in dimensions between teacher and student models. Besides, MobileBERT requires its student model to have the same number of layers as its teacher. MiniLM [17] and DynaBERT [18] allow more flexibility for the number of layers and hidden dimension size of the student. MiniLM proposes a deep self-attention distillation of the last layer of the teacher model. However, it lacks proper guidelines for constructing and initializing the student model. DynaBERT constructs and initializes a student model by taking a sub-network of the teacher network and reusing its parameter value. However, its training process is complicated as it involves rewiring the teacher network and requires two stages of knowledge distillation, which increase the computational cost and training time. Pea-KD [19] proposes a compressed structure of the encoder with shared layers and shuffled query and key matrices to enlarge an insufficient capacity of the student model. It also proposes an initialization method for the student model. It generates four new labels for a given binary classification task using the teacher’s predictions. It initializes the student model by pre-training it with those new labels to encourage it to learn the teacher’s high-level knowledge. During pre-training, the student model of Pea-KD uses a classification layer with an output size of four to classify the input into those four generated labels. In the KD process, the student model has to classify an input into one of the binary classes of the original task. Thus, it changes its pre-trained classification layer to a smaller output size of two. In other words, Pea-KD does not use the entire pre-trained parameters and replaces the last classification layers with non-pre-trained ones. Pea-KD has limitations in that it pre-trains extra parameters, and does not initialize the exact classification layer used in the KD process.

#### Knowledge Distillation on Transformer decoders

KD on GPT falls into this category. As mentioned in the introduction, fewer works in this category have been proposed compared to Knowledge Distillation on encoders. KnGPT2 [33] compresses the embedding and Transformer layers of GPT-2 using Kronecker decomposition. It uses KD to compensate for the performance drop of the compressed model. However, lots of computation cost occurs during decomposition. CAN [34] constructs a student model with compressed self-attention and cross-attention using simplified matrix multiplication. It shows an accuracy gain when used with KD. Although it proposes a compression method for the Transformer neural machine translation model consisting of an encoder and decoder, it only compresses the decoder. Moreover, it makes the encoder deeper (from 6 to 12 layers) to compensate for the accuracy loss caused by reducing the size of the decoder. Therefore, there remains room for improvement by compressing the entire model.

#### Knowledge Distillation on Transformer encoders and decoders

Weight Distillation [35] transfers knowledge in parameters of the teacher model through a special module named parameter generator. The parameter generator generates parameters of the student model by weight grouping and projection. Weight Distillation consists of two phases: generating parameters of the student model using the parameter generator and training the generated student model. It requires training both the parameter generator and student model sequentially. Therefore, twice the training cost is inevitable. On the other hand, PET does not use the additional parameters for generating a smaller model and maximizes its training efficiency by training only the exact parameters of the target compression model. Once a target compression model is generated, PET does not transform its structure or replace its components.

### Parameter sharing

Parameter sharing is a widely used technique to reduce the size of the Transformer. It shares the same sets of parameters across multiple parts of the model. Universal Transformer [36] shares parameters of one layer across all the layers with a dynamic halting mechanism and achieves better performance than the vanilla Transformer. However, it increases the size of the one layer. As a result, the number of parameters of the vanilla Transformer-base model and the compressed one are the same. It also takes more computation time for each layer and fails to gain computational efficiency [37]. Takase and Kiyono [37] improved the speed of the Universal Transformer by compressing an *M*-layered Transformer model with *N* layers of parameters, where 1 < *N* < *M*. However, those previous parameter sharing-based compression methods cannot exploit the knowledge of large pre-trained models.

### Other compression techniques

Pruning, quantization, and matrix decompositions are widely known compression methods. Unstructured pruning [38] and quantization [39] reduce the memory usage of models by removing redundant parameters and representing model weights with fewer bits, respectively. However, these methods require hardware or acceleration libraries to achieve speedup gains. Structured pruning [26, 40] reduces attention heads and layers in the Transformer, while matrix decomposition [41, 42] is efficient for reducing parameters in embedding, feedforward, and attention layers. MDN [28] unifies existing compression techniques and improves Transformer inference speed, utilizing several training strategies to compensate for accuracy loss resulting from model size reduction. These techniques are orthogonal to our work; combining them with our work to achieve more compression and performance enhancement is a future work.

## Proposed method

We propose PET, an efficient Transformer compression method which improves the conventional Knowledge Distillation (KD) process. Given an NLP task and a large pre-trained Transformer model for the task, PET returns a compact Transformer model which is small and fast while preserving the performance of the large model. PET proposes a parameter-efficient student architecture and an effective initialization method for KD on both the encoder and the decoder. We first provide an overview of the main challenges for the problem and our main ideas to tackle them. We then describe each idea in detail.

The main challenges and ideas of PET are as follows:

(1) **How can we efficiently compress the encoder and the decoder?** We find *replaceable pairs* of the modules in the encoder and the decoder, respectively, considering their characteristics. The *replaceable pairs* of the modules share their parameters to construct a small student model.
(2) **How can we initialize the weights of the student model to maximize the performance of a given task?** PET warms up the student model with a simplified task before the KD process. The simplified task is an easier classification task to initialize the student model which has less number of the classes than the original translation task in KD.
(3) **How can we maximize the effect of the proposed warming up process?** We carefully design the mapping of prediction probabilities to labels of the student model for the simplified task. The student model utilizes the pre-trained knowledge effectively during KD, since the proposed warming up process does not require any modification to the model structure though the number of classes for warming up and translation are different.

The overall process of PET is as follows: First, we construct a smaller student model which shares the parameters of the replaceable pairs within the encoder and decoder. Second, we warm up the student model with a simplified task for a few epochs. Lastly, we train the student model with the original task using KD. The last step of learning the original task follows the conventional KD process, which involves using a training loss that combines the task loss and the KL divergence loss of the teacher and student models’ outputs. We describe the details of the first and second processes in the following.

### Finding replaceable pairs in encoder and decoder

Voita et al. [43] demonstrate that parameters have different sensitivities for pruning depending on layer order and types of multi-head attention. We assume that the same applies to parameter sharing, and how we make pairs of parameters to share considerably affects accuracy. We aim to identify pairs of layers and matrices in multi-head attention (query, key, and value), insensitive to parameter sharing; we denote these pairs as *replaceable pairs*. In the following, we describe how we find replaceable pairs to construct a four-layer Transformer model with the same number of parameters as two layers.

#### Layer-wise replaceable pairs

We first examine the impact of parameter sharing across layers and pairing rules. We compare BLEU [44] scores of several four-layer Transformer models with different sets of layer-wise pairs: {(*L*_1_, *L*_2_), (*L*_3_, *L*_4_)}, {(*L*_1_, *L*_3_), (*L*_2_, *L*_4_)}, and {(*L*_1_, *L*_4_), (*L*_2_, *L*_3_)}. We then take the best set and share the parameters of the layers belonging to the same layer-wise pair. Table 2 shows the results. Note that all the models have the same number of parameters in each layer. Models with layer-wise pairs from {(*L*_1_, *L*_2_), (*L*_3_, *L*_4_)} and {(*L*_1_, *L*_3_), (*L*_2_, *L*_4_)} outperform the vanilla Transformer model (No pair), although the vanilla model has twice the number of parameters. It shows that the Transformer model is over-parameterized and layer-wise parameter sharing is effective to relieve the redundancy.

**Table 2:** BLEU scores according to different layer-wise pairs. No pair denotes a four-layer vanilla Transformer model without layer-wise and matrix-wise pairs. Layer-wise parameter-shared model with pairs from {(*L*_1_, *L*_3_), (*L*_2_, *L*_4_)} shows the best accuracy with half the size of the four-layer vanilla Transformer model.

Set of layer-wise pairs	Ratio of #Parameters	BLEU
No pair	1	34.24
{(*L*_1_, *L*_2_), (*L*_3_, *L*_4_)}	0.5	34.40
{(*L*_1_, *L*_3_), (*L*_2_, *L*_4_)}	0.5	34.57
{(*L*_1_, *L*_4_), (*L*_2_, *L*_3_)}	0.5	33.83

#### Matrix-wise replaceable pairs

We find matrix-wise pairs that best fit with layer-wise replaceable pairs, (*L*_1_, *L*_3_) and (*L*_2_, *L*_4_). We compare BLEU scores of four-layer Transformer models with those layer-wise pairs changing sets of matrix-wise pairs. We make pairs of query, key, and value matrices in multi-head attention sub-layers as follows: (query, key), (key, value), and (value, query).

**Fig 2:**
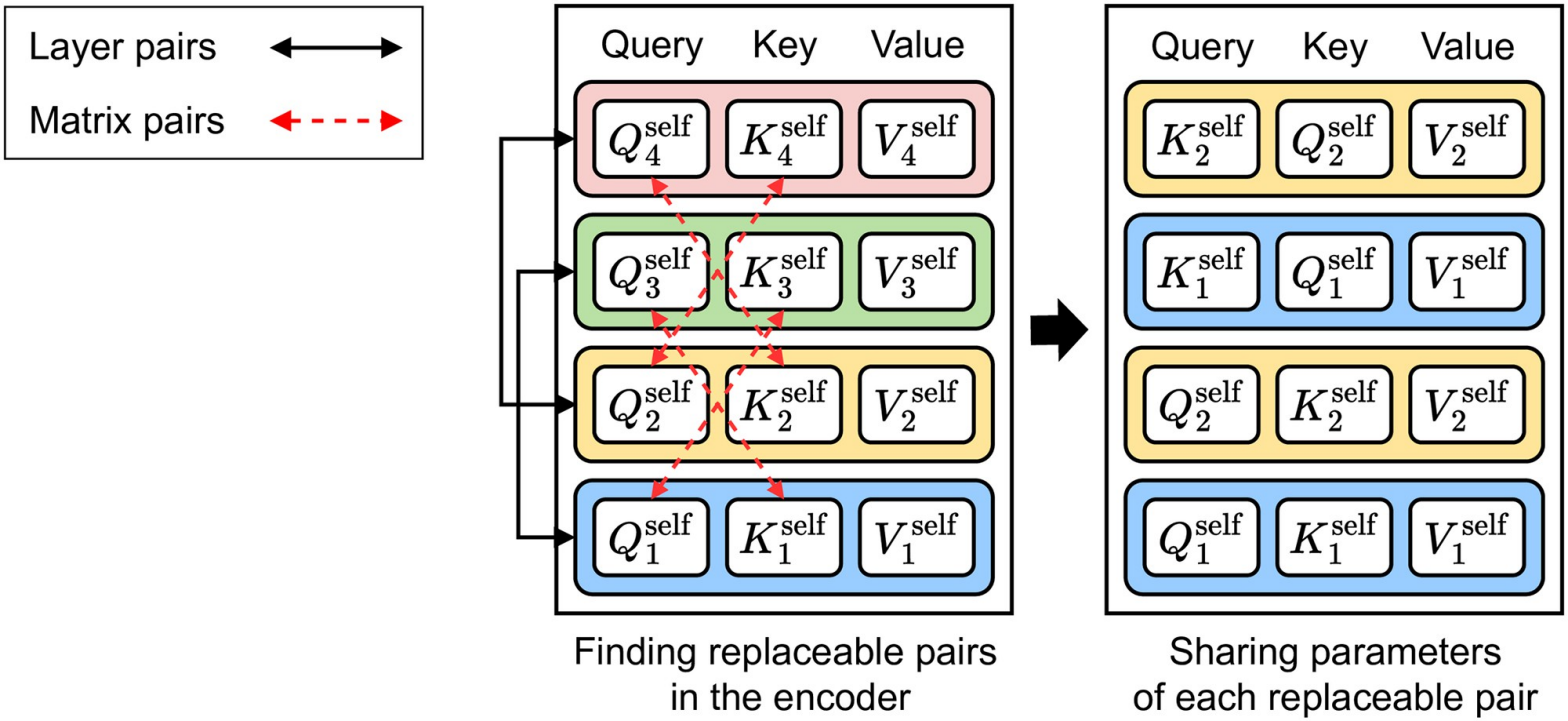
Finding replaceable pairs in the encoder. $Q_{i}^{\text{self}}$ ,$K_{i}^{\text{self}}$, and $V_{i}^{\text{self}}$ are query, key, and value matrices of the self-attention in the *i*-th encoder layer, respectively. We generate a compressed model (right) by sharing the weights of replaceable pairs in the uncompressed model (left).

We first apply those patterns of matrix-wise pairs to self-attention sub-layers in the encoder. Table 3 shows how the BLEU score changes according to different matrix-wise pairs in the encoder. The matrix-wise pairs from $\{(Q_{1}^{\text{self}}$, $K_{3}^{\text{self}}),(Q_{2}^{\text{self}}$, $K_{4}^{\text{self}})\}$ enhance the performance of the model the most. Thus, we make pairs with query and key matrices from different layers in the encoder. In Fig 2, $Q_{3}^{\text{self}},K_{3}^{\text{self}},Q_{4}^{\text{self}}$, and $K_{4}^{\text{self}}$ are replaced by their paired matrices, $K_{1}^{\text{self}},Q_{1}^{\text{self}},K_{2}^{\text{self}}$, and $Q_{2}^{\text{self}}$, respectively.

**Table 3:** BLEU scores according to different matrix-wise pairs in the encoder. Matrix-wise pairs from $\left\{\left(Q_{1}^{\text{self}},K_{3}^{\text{self}}\right),\left(Q_{2}^{\text{self}},K_{4}^{\text{self}}\right)\right\}$ improve the performance of Transformer the most.

Set of matrix-wise pairs	Ratio of #Parameters	BLEU
No pair	1	34.24
Only layer-wise pairs	0.5	34.57
$\{(Q_{1}^{\text{self}}$ , $K_{3}^{\text{self}}),(Q_{2}^{\text{self}}$, $K_{4}^{\text{self}})\}$	0.5	34.62
$\{(K_{1}^{\text{self}}$ , $V_{3}^{\text{self}}),(K_{2}^{\text{self}}$, $V_{4}^{\text{self}})\}$	0.5	34.35
$\{(V_{1}^{\text{self}}$ , $Q_{3}^{\text{self}}),(V_{2}^{\text{self}}$, $Q_{4}^{\text{self}})\}$	0.5	34.51

**Fig 3:**
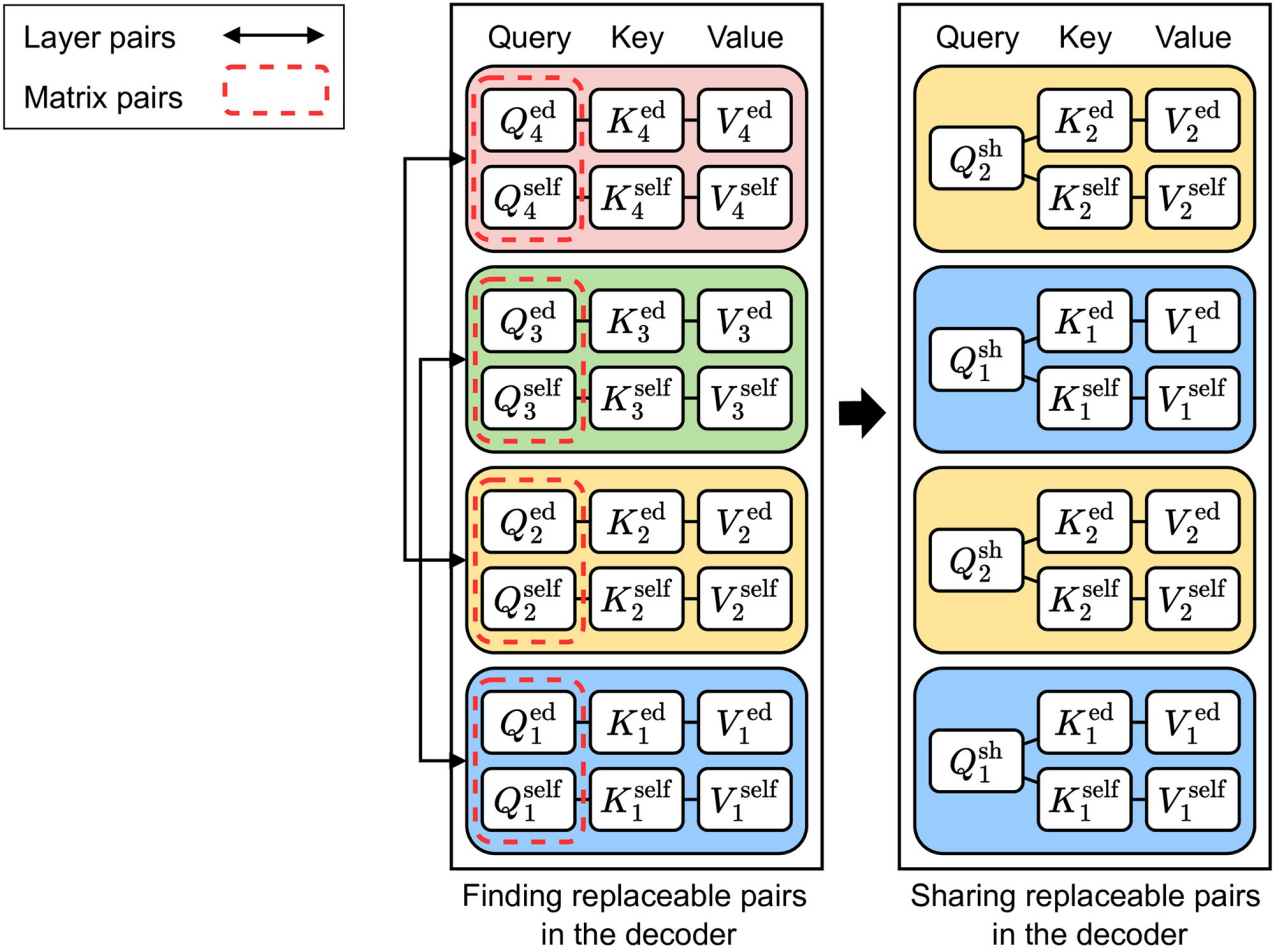
Finding replaceable pairs in the decoder. $Q_{i}^{\text{self}}$ ,$K_{i}^{\text{self}}$, and $V_{i}^{\text{self}}$ are query, key, and value matrices of the self-attention sub-layer in the *i*-th decoder layer, respectively. Similarly, $Q_{i}^{\text{ed}}$,$K_{i}^{\text{ed}}$, and $V_{i}^{\text{ed}}$ are those of the encoder-decoder attention sub-layer in the *i*-th decoder layer, respectively. We share the parameters of the replaceable pairs from $\left\{\left(L_{1},L_{3}\right),\left(L_{2},L_{4}\right),\left(Q_{i}^{\text{self}},Q_{i}^{\text{ed}}\right)\right\}$ in the uncompressed model (left) to generate a compressed model (right). The shared matrix of $Q_{i}^{\text{self}}$ and $Q_{i}^{\text{ed}}$ is denoted as $Q_{i}^{\text{sh}}$.

We then apply matrix-wise pairs of the same pairing patterns we use in Table 3 to self-attention and encoder-decoder attention sub-layers in the decoder. We utilize four-layer Transformer models where layer-wise replaceable pairs are (*L*_1_, *L*_3_) and (*L*_2_, *L*_4_), and matrix-wise replaceable pairs in the encoder are $(Q_{1}^{\text{self}}$, $K_{3}^{\text{self}})$ and $(Q_{2}^{\text{self}}$, $K_{4}^{\text{self}})$ Table 4 shows how the BLEU scores change according to different matrix-wise pairs in the decoder. When comparing Tables 3 and 4, the same matrix-wise pairing patterns perform differently depending on whether they are used only in the encoder, or in both the encoder and decoder. All types of matrix-wise pairs drop the BLEU scores when used in the decoder. We achieve less accuracy drop when we apply only matrix-wise pairs to the self-attention sub-layers excluding the encoder-decoder attention ones. We conclude that parameters in the decoder are more sensitive to parameter sharing and this is mainly due to the encoder-decoder attention sub-layers. As mentioned in the Related work, the key and value in the encoder-decoder self-attention come from the encoder outputs, which encode the source sequence, while the query is from the previous decoder states related to the target sequence. We need different matrix-wise pairs in the decoder. Our solution is to share the query matrices in self-attention and encoder-decoder attention because both process the decoder outputs. Sharing those matrices has one more strength, compared to the case in the encoder: it reduces the number of parameters. We achieve 0.47x smaller model with a 34.51 BLEU score in the same experiment in Table 4. We select those query matrices as matrix-wise replaceable pairs in the decoder. Fig 3 illustrates the replaceable pairs in the decoder. We reduce the number of parameters of the original model (left) by applying weight-sharing for replaceable pairs of layers and matrices (right). The parameters of the first and second layers replace the parameters of the third and fourth layers, respectively. Also, the parameters of the query matrices of the self-attention and encoder-decoder attention are shared in each layer.

**Table 4:** BLEU scores according to different matrix-wise pairs in the decoder. Matrix-wise pairs perform differently in the decoder, unlike in the encoder. Specifically, matrix-wise pairs of the encoder-decoder-attention in the decoder degrade the BLEU scores significantly.

Set of matrix-wise pairs	Ratio of #Parameters	BLEU
No pair	1	34.24
Only layer-wise pairs	0.5	34.57
$\{(Q_{1}^{\text{self}}$ , $K_{3}^{\text{self}}),(Q_{2}^{\text{self}}$, $K_{4}^{\text{self}}),(Q_{1}^{\text{ed}}$, $K_{3}^{\text{ed}}),(Q_{2}^{\text{ed}}$, $K_{4}^{\text{ed}})\}$	0.5	5.13
$\{(K_{1}^{\text{self}}$ , $V_{3}^{\text{self}}),(K_{2}^{\text{self}}$, $V_{4}^{\text{self}}),(K_{1}^{\text{ed}}$, $V_{3}^{\text{ed}}),(K_{2}^{\text{ed}}$, $V_{4}^{\text{ed}})\}$	0.5	1.88
$\{(V_{1}^{\text{self}}$ , $Q_{3}^{\text{self}}),(V_{2}^{\text{self}}$, $Q_{4}^{\text{self}}),(V_{1}^{\text{ed}}$, $Q_{3}^{\text{ed}}),(V_{2}^{\text{ed}}$, $Q_{4}^{\text{ed}})\}$	0.5	1.86
$\{(Q_{1}^{\text{self}}$ , $K_{3}^{\text{self}}),(Q_{2}^{\text{self}}$, $K_{4}^{\text{self}})\}$	0.5	23.78
$\{(K_{1}^{\text{self}}$ , $V_{3}^{\text{self}}),(K_{2}^{\text{self}}$, $V_{4}^{\text{self}})\}$	0.5	34.00
$\{(V_{1}^{\text{self}}$ , $Q_{3}^{\text{self}}),(V_{2}^{\text{self}}$, $Q_{4}^{\text{self}})\}$	0.5	34.16

### Warming up with a simplified task

To initialize the student that performs well to a challenging task efficiently, we propose to pre-train the student model with a simplified task. Sequence-to-sequence tasks can be reduced to a multi-classification task, classifying each token over the target vocabulary. These tasks usually have a lot of classes; e.g., in our case, the student model has to classify each token into 3-40K classes. It is too challenging for a student model with a small number of parameters to handle that many classes. With more classes, the student model has to learn more complex decision boundaries using its small learning capacity. We warm up the student model for KD and its complicated task by making it learn the knowledge step by step, starting with a much easier task and then moving on to solve difficult tasks. The proposed easier task is a simplified version of the original task of KD. The general knowledge learned from the easier task helps the student effectively solve the harder task.

The proposed simplified classification task has only four classes to relieve the complexity of the task. We define new labels for the pre-training dataset using the teacher model’s prediction. We classify the teacher’s prediction *f*_*t*_(*x*)_*i*_ of each token *i* into four classes: 1) confidently correct, 2) correct but not confident, 3) confidently wrong, and 4) wrong but not confident. We describe the detailed rule for assigning a label for each data instance in Table 5. The student model is trained classifying the teacher model’s prediction for the *i*-th token *f*_*t*_(*x*)_*i*_ into those labels, where *f*_*t*_ is the teacher model, and *x* is the input sentence.

**Table 5:** The label assignment rule for the teacher model’s predictions. *y*_*i*_ is the target label for the token *x*_*i*_. *ϵ*_1_ and *ϵ*_2_ are hyper-parameters regarding the model’s confidence for the correct and wrong inferences, respectively.

Class	Condition
Confidently correct	argmax(*f*_*t*_(*x*)_*i*_) = *y*_*i*_ and max(*f*_*t*_(*x*)_*i*_) ≥ *ϵ*_1_
Correct, but not confident	argmax(*f*_*t*_(*x*)_*i*_) = *y*_*i*_ and max(*f*_*t*_(*x*)_*i*_) ≤ *ϵ*_1_
Confidently wrong	argmax(*f*_*t*_(*x*)_*i*_) ≠ *y*_*i*_ and max(*f*_*t*_(*x*)_*i*_) ≥ *ϵ*_2_
Wrong, but not confident	argmax(*f*_*t*_(*x*)_*i*_) ≠ *y*_*i*_ and max(*f*_*t*_(*x*)_*i*_) ≤ *ϵ*_2_

The initialization method of PET can be viewed as a variation of the initialization method of Pea-KD [19]. However, there are two differences between PET and Pea-KD in terms of targets for labeling and computing loss, and what they pursue in the initialization process. First, they assign labels and compute loss on different targets; a sequence vs. tokens. BERT models used in Pea-KD predict the label of a sequence; they make a prediction probability for input [CLS] tokens. We evaluate whether the teacher model is correct or not based on the prediction to a sequence. Therefore, Pea-KD assigns a label to a sequence and sums up losses generated from each sequence. The student model learns how well the teacher model predicts each sentence and how complicate it is to classify each sentence in the initialization process. However, Transformer decoder used in PET generates the prediction probabilities for each token. PET assigns labels to tokens and sums up losses generated from each token. The student model learns how well the teacher model generates each token and how hard it is to generate each token in the initialization process.

Second, they use the pre-training tasks in an opposite point of view. PET addresses multi-class classification tasks which are too complicated for the student model. Thus, the goal of a pre-training task in PET is to simplify the difficult tasks into easier ones by reducing the number of classes from large to small. However, Pea-KD [19] addresses binary classification tasks where the student gets too simple information for the student to be trained with. Therefore, it enlarges the number of classes giving more information at the pre-training step. Although both methods aim to enhance the performance of the KD by efficient initialization of the student model, PET relieves the student with fewer classes, whereas Pea-KD [19] burdens the model with more information.

### Mapping prediction probabilities to labels of simplified task

**Fig 4:**
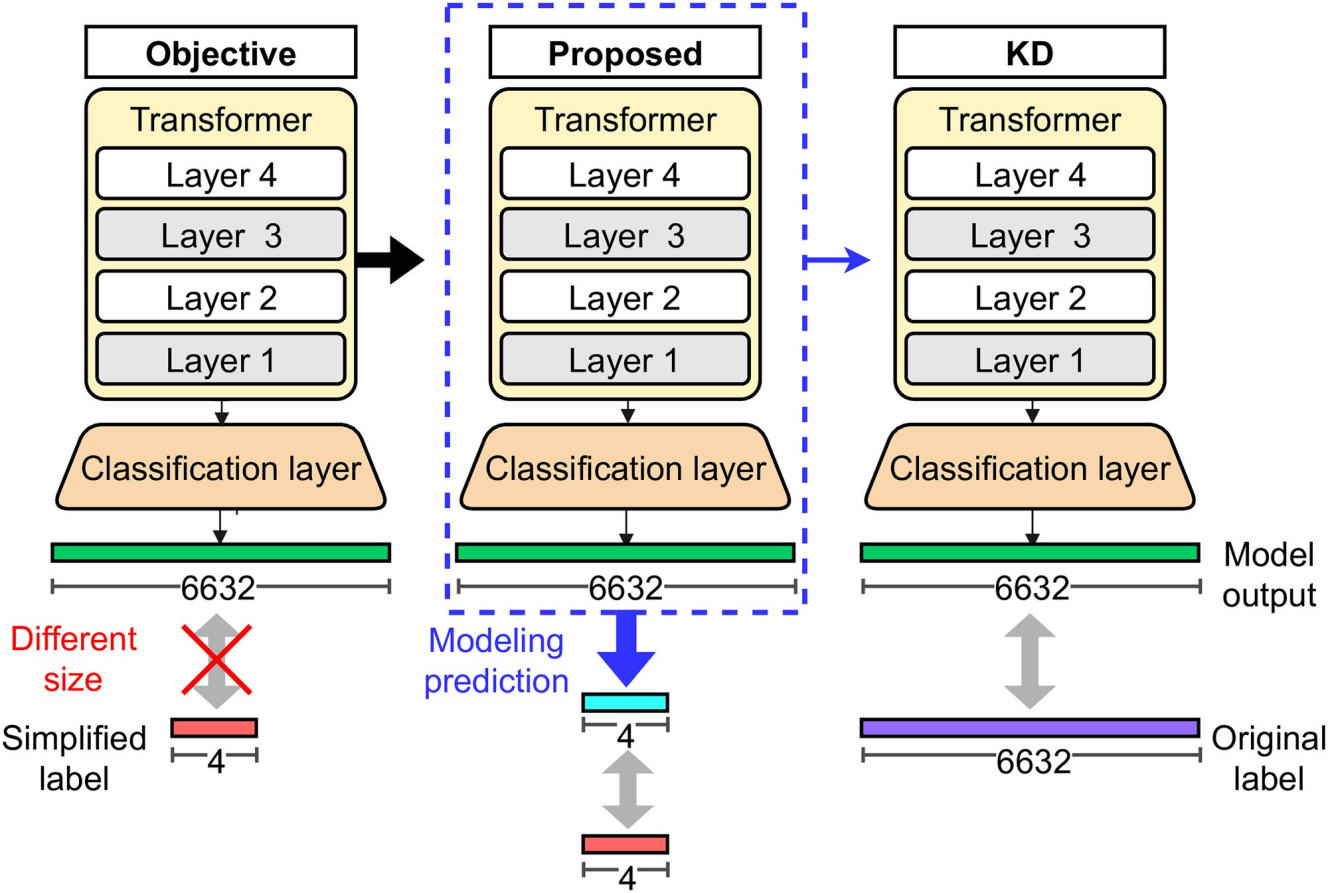
Mapping predictions of the model to the simplified labels. The number of simplified labels is much smaller than the output size of the model (in this case, 4 vs. 6632). Instead of modifying the model’s structure to match the output size of the model with the size of the simplified labels, we map predictions of the model to the simplified labels. As we do not modify the structure, we use the same parameters in the KD process.

There is an additional challenge to warm up the student model using the proposed simplified task. The output size of the student model does not match the number of classes because we reduce it to four. Fig 4 shows this mismatch. The size of the output vector of the leftmost model is 6632. As mention in the Related Works, the Transformer model returns prediction probabilities for the number of classes equal to the size of the output vector. Therefore, we only get the prediction probabilities for 6632 classes, not four classes. To train the student model with the proposed simplified task of classifying the generated four labels, we need prediction probabilities for the four classes. That is, an output vector of size four from the student model is required. There are two naïve approaches to solve this challenge: reducing the output size of the model from the number of target classes to four or adopting an additional projection layer that maps the output vector of the model into four-dimensional space. These two naïve approaches change the size of the final output of the model and make it possible to obtain the model’s prediction probabilities for the reduced number of classes. However, they still have limitations in that they change the structure of the model by reducing the output layer size or applying an additional layer. They fail to efficiently leverage the pre-trained parameters in KD since they use different model structures for pre-training and KD. To overcome the limitation of the naïve approaches, we carefully design the prediction probabilities to the proposed four classes in Table 5. As a result, we train the pre-trained student model without any modification in its structure in KD.

We assume that P(confident) and P(correct) are independent. Then, the prediction probabilities for the proposed labels are as follows:

$$\begin{array}{c} \begin{array}{cc} P(\text{confidently}\;\text{correct}) & =P(\text{confident})P(\text{correct})\\ P(\text{correct},\;\text{but}\;\text{not}\;\text{confident}) & =P(\text{not}\;\text{confident})P(\text{correct})\\ P(\text{confidently}\;\text{wrong}) & =P(\text{confident})P(\text{not}\;\text{correct})\\ P(\text{wrong},\;\text{but}\;\text{not}\;\text{confident}) & =P(\text{not}\;\text{confident})P(\text{not}\;\text{correct}) \end{array} \end{array}$$ (2)

We define P(confident), P(notconfident), P(correct), and P(notcorrect) as follows:

$$\begin{array}{c} \begin{array}{cc} P(\text{confident}) & =\text{max}(f_{s}{\left(x\right)}_{i})\\ P(\text{correct}) & =f_{s}{\left(x\right)}_{y_{i}}\\ P(\text{not}\;\text{confident}) & =1-\text{max}(f_{s}{\left(x\right)}_{i})\\ P(\text{not}\;\text{correct}) & =1-f_{s}{\left(x\right)}_{y_{i}} \end{array} \end{array}$$ (3)

where *f*_*s*_(*x*)_*i*_ is the predicted probability vector of the *i*-th token in the student model *f*_*s*_, and *x* is the input sentence. $f_{s}{\left(x\right)}_{y_{i}}$ is the probability score of the ground truth label for the *i*-th token.

Then, we get prediction probabilities for each label as follows:

$$\begin{array}{c} \begin{array}{cc} P(\text{confidently}\;\text{correct}) & =\text{max}\left(f_{s}{\left(x\right)}_{i}\right)f_{s}{\left(x\right)}_{y_{i}}\\ P(\text{correct},\;\text{but}\;\text{not}\;\text{confident}) & =\left\{1-\text{max}\left(f_{s}{\left(x\right)}_{i}\right)\right\}f_{s}{\left(x\right)}_{y_{i}}\\ P(\text{confidently}\;\text{wrong}) & =\text{max}\left(f_{s}{\left(x\right)}_{i}\right)\left\{1-f_{s}{\left(x\right)}_{y_{i}}\right\}\\ P(\text{wrong},\;\text{but}\;\text{not}\;\text{confident}) & =\left\{1-\text{max}\left(f_{s}{\left(x\right)}_{i}\right)\right\}\left\{1-f_{s}{\left(x\right)}_{y_{i}}\right\} \end{array} \end{array}$$ (4)

The motivation behind the definitions of $P\left(\text{confident}\right)=\text{max}(f_{s}{\left(x\right)}_{i})$ is that a confident model will predict the answer token with high probability while assigning low probabilities to other tokens in the vocabulary. For $P\left(\text{correct}\right)=f_{s}{\left(x\right)}_{y_{i}}$, the probability of the model being correct will increase if the model assigns a high probability to the ground truth token while assigning low probabilities to the others.

As we do not modify the structure of the model in pre-training, it becomes possible to directly use the same structure in KD without any modification. The rightmost figure of Fig 4 shows this. PET does not require an additional process for KD, such as replacing the classification layer or detaching the projection layer as naïve approaches do.

### Computational cost of PET

PET compresses both the depth and width of the model (i.e., the number and dimension of layers). When the depth-wise and width-wise compression factors are *c*_*d*_ and *c*_*w*_, respectively, the computational complexity of the model is reduced as follows:

- Attention modules
  $$\begin{array}{c} L\cdot O\left(T^{2}\cdot D\right)\to \frac{L}{c_{d}}\cdot O\left(T^{2}\cdot \frac{D}{c_{w}}\right) \end{array}$$ (5)
- Feed-forward modules
  $$\begin{array}{c} L\cdot O\left(T\cdot D^{2}\right)\to \frac{L}{c_{d}}\cdot O\left(T\cdot \frac{D^{2}}{c_{w}^{2}}\right) \end{array}$$ (6)

where *L*, *T*, and *D* denote the number of layers, input sequence length, and layer dimension of the baseline model, respectively.

The training process of PET is also computationally efficient because it does not introduce extra parameters or intermediate models, unlike previous KD works [7, 15]. Moreover, the additional computational cost incurred by the warming up process is negligible as it pre-trains the student model for only a few epochs.

## Experiments and discussion

We perform experiments to answer the following questions:

Q1 **Translation accuracy**. How accurate is PET compared to the competitors?
Q2 **Translation speed**. How fast is PET compared to the competitors?
Q3 **Effectiveness of replaceable pairs**. Do the proposed replaceable pairs of modules outperform other weight-sharing patterns?
Q4 **Effectiveness of warming up with simplified task**. Does warming up before training improve the performance of the model?
Q5 **Effectiveness of mapping of prediction probabilities to labels in simplified task**. Does the proposed mapping of prediction probabilities to labels of simplified task improve the performance of the model?
Q6 **Sensitivity analysis**. How much does the beam size affect the accuracy of PET? How robust is PET to random trials?

### Experimental settings

#### Dataset

We evaluate PET on neural machine translation tasks with real-world datasets: IWSLT’14 English↔German (EN↔DE), English↔Spanish (EN↔ES), IWSLT’17 English↔French (EN↔FR), WMT’17 English↔Finnish (EN↔FI), and English↔Latvian (EN↔LV). We summarized the statistics of the datasets in Table 6. We get the IWSLT’14 English↔German dataset from FAIRSEQ [45], the other IWSLT datasets from the online corpus WIT3 [46], and the WMT datasets from their official website. We preprocess them following FAIRSEQ.

**Table 6:** Summary of the datasets.

Dataset	Language	Classes	Sentences	Tokens
IWSLT’14 DE↔EN^1^	DE	8.85K	1.60M	40.36M
	EN	6.63K	1.60M	39.49M
IWSLT’14 ES↔EN^2^	ES	8.50K	1.69M	41.70M
	EN	6.81K	1.69M	41.92M
IWSLT’17 FR↔EN^2^	FR	8.44K	2.37M	67.69M
	EN	7.18K	2.37M	60.72M
WMT’17 FI↔EN^3^	FI	35.39K	2.23M	52.65M
	EN	19.62K	2.23M	60.52M
WMT’17 LV↔EN^3^	LV	39.39K	3.96M	57.19M
	EN	23.57K	3.96M	63.05M

^1^ http://dl.fbaipublicfiles.com/fairseq/data/iwslt14

^2^ https://wit3.fbk.eu

^3^ https://data.statmt.org/wmt17/translation-task/preprocessed

#### Competitors

We compare PET with two competitors: Patient KD [29] and CAN [34]. Patient KD improves on the original KD [21] by distilling intermediate representations in addition to the classification probabilities. They minimize the mean squared error between intermediate representations of student and teacher models to make the student model mimic the teacher model. On the other hand, CAN is a state-of-the-art Transformer compression algorithm that accelerates the inference speed by integrating sublayers in the decoder. CAN has a deep encoder and shallow decoder to maximize its inference speed for autoregressive tasks.

The comparison between PET and these algorithms aims to show the superiority of PET over existing KD and Transformer compression algorithms.

#### Compression target

We use the Transformer-base [1] model for comparison. We implement PET and competitors based on the open-source implementation [45] of the Transformer model. We use the same configuration of hyper-parameters as in the original paper [1] for WMT translation tasks; the number of encoder and decoder layers, embedding size, the number of heads, and feed-forward network’s hidden size are 6, 512, 8, and 2048, respectively. We use a smaller Transformer model for IWSLT following the configuration of transformer_iwslt_de_en in [45]. We change the number of heads and hidden size of the feed-forward network to 4 and 1024, respectively.

#### Training details

We construct a four-layer student model and share the parameters between layer-wise replaceable pairs. As a result, our student model has the same number of parameters with a two-layer model. Also, we reduce layer dimensions in the encoder and decoder from (512, 512) to (320, 240) when the target language is English and (240, 320) when the source language is English. We set the dimension of the FFN layer to be twice of that of the attention layer. We warm up the student model for three epochs and then train it with the original task following the conventional KD process. Our implementation is developed on the open-source library of the Transformer model, FAIRSEQ. We use the default training configurations of the library and stop training when the model stops improving on the validation set over three epochs. We report the average test BLEU score for three runs with a beam size of 5.

#### Evaluation

We use BLEU [44] score and sentence throughput for comparison. The BLEU score measures the translation accuracy while the sentence throughput measures the inference speed of the model by representing the number of sentences processed per second. We compare PET and its competitors at various compression rates in terms of the number of parameters excluding the embedding layers. All of our experiments are performed on a single machine with a GeForce RTX 3090 GPU.

### Translation accuracy (Q1)

**Fig 5:**
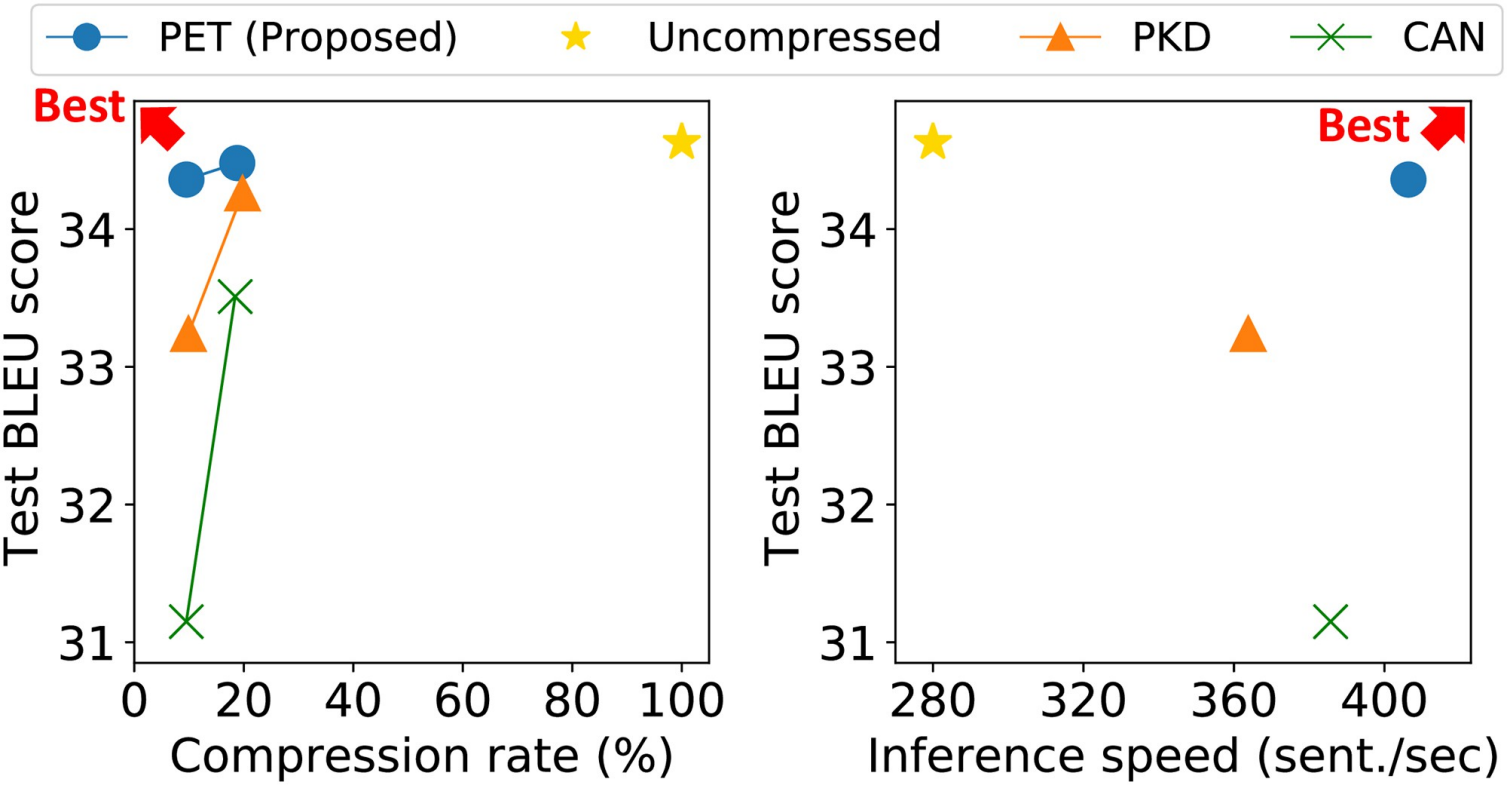
Trade-off of accuracy, compression rate, and speed on IWSLT’14 DE→EN. PET shows the best trade-off between accuracy and compression rate. Also, PET gives the best trade-off between accuracy and inference speed.

We first compare the translation accuracy of PET and competitors on various datasets and compression rates. In all cases, PET has a slightly smaller number of parameters than competitors for a thorough comparison. The leftmost figure of Fig 5 shows the experimental results on IWSLT’14 DE-EN. PET reduces the size of the Transformer-base model to 9.52% with a slight reduction in the BLEU score (0.27) while outperforming larger competitors. Table 7 shows the comparison results on the diverse language pairs of datasets. PET shows the highest BLEU score with even a smaller number of parameters in all language pairs. In summary, PET is more accurate and memory-efficient than competitors.

**Table 7:** Comparison of the BLEU score. The best method is in bold. ΔBLEU denotes the difference between the uncompressed model and the compressed model. PET achieves the highest BLEU score with a smaller model size on all datasets.

Task	Model	Compression rate (%)	BLEU	ΔBLEU
IWSLT’14 DE→EN	Uncompressed	100	34.63	-
	Patient-KD	9.88	33.24 ± 0.23	1.39
	CAN	9.52	31.15 ± 0.39	3.48
	**PET (proposed)**	**9.51**	**34.36 ± 0.14**	**0.27**
IWSLT’14 EN→DE	Uncompressed	100	28.53	-
	Patient-KD	9.60	26.50 ± 0.14	2.03
	CAN	9.90	26.42 ± 0.21	2.11
	**PET (proposed)**	**9.50**	**27.95 ± 0.18**	**0.58**
IWSLT’14 ES→EN	Uncompressed	100.00	39.44	-
	Patient-KD	13.55	38.22 ± 0.11	1.22
	CAN	13.99	37.77 ± 0.73	1.67
	**PET (proposed)**	**13.22**	**38.52 ± 0.61**	**0.92**
IWSLT’14 EN→ES	Uncompressed	100.00	36.12	-
	Patient-KD	16.75	34.73 ± 1.15	1.39
	CAN	16.67	34.43 ± 1.13	1.69
	**PET (proposed)**	**16.18**	**35.31 ± 1.18**	**0.81**
IWSLT’17 FR→EN	Uncompressed	100.00	39.16	-
	Patient-KD	13.81	37.97 ± 0.09	1.19
	CAN	14.30	37.34 ± 0.09	1.82
	**PET (proposed)**	**13.48**	**38.31 ± 0.06**	**0.85**
IWSLT’17 EN→FR	Uncompressed	100.00	39.55	-
	Patient-KD	17.00	38.69 ± 0.11	0.86
	CAN	16.90	38.31 ± 0.15	1.24
	**PET (proposed)**	**16.43**	**38.70 ± 0.28**	**0.85**
WMT’17 FI→EN	Uncompressed	100.00	23.45	-
	Patient-KD	18.86	18.28 ± 0.13	5.17
	CAN	18.58	18.23 ± 0.17	5.22
	**PET (proposed)**	**18.58**	**19.06 ± 0.47**	**4.39**
WMT’17 EN→FI	Uncompressed	100.00	15.76	-
	Patient-KD	17.72	12.99 ± 0.12	2.77
	CAN	17.50	13.03 ± 0.96	2.73
	**PET (proposed)**	**17.50**	**13.38 ± 0.49**	**2.38**
WMT’17 LV→EN	Uncompressed	100.00	19.90	-
	Patient-KD	20.61	16.48 ± 0.04	3.42
	CAN	20.63	15.96 ± 1.12	3.94
	**PET (proposed)**	**20.35**	**17.59 ± 0.09**	**2.31**
WMT’17 EN→LV	Uncompressed	100.00	17.04	-
	Patient-KD	13.56	14.29 ± 0.06	2.75
	CAN	16.57	14.46 ± 1.56	2.59
	**PET (proposed)**	**13.08**	**15.55 ± 0.24**	**1.49**

### Translation speed (Q2)

We compare the translation speed of PET and competitors on IWSLT’14 DE↔EN dataset in the rightmost figure of Fig 5. PET shows the fastest performance processing 45.15% more sentences than the uncompressed model with only 0.27%p of accuracy drop. Also, PET shows the best accuracy-speed tradeoff, closest to the best point with the highest accuracy and the fastest inference speed.

### Effectiveness of replaceable pairs (Q3)

We evaluate the performance with different weight-sharing patterns on IWSLT’14 DE-EN to verify the effect of the replaceable pairs on the model accuracy. Table 3 shows the experimental results for the replaceable pairs in the encoder. Our proposed replaceable pair, *Q* and *K*, shows the highest BLEU score. Table 8 shows the experimental results for the decoder. Our proposed replaceable pair, *Q*^self^ and *Q*^ed^, achieves the best BLEU score with the same number of parameters as others. Note that we have a significant performance degradation when we share *Q*^self^ and *K*^self^. This result comes from the difference between quires and keys in the decoder; queries come from the previous decoder layer and keys come from the encoder. PET avoids the degradation by carefully considering the characteristics of each module.

**Table 8:** Effect of the replaceable pairs. The accuracy of the model changes according to the weight-sharing patterns, and the proposed replaceable pairs achieve the highest BLEU score.

Set of pairs	BLEU
{(*Q*^self^, *Q*^ed^)} (proposed)	34.36
{(*Q*^self^, *K*^self^)}	30.94
{(*K*^self^, *V*^self^), (*K*^ed^, *V*^ed^)}	33.98

### Effectiveness of warming up with simplified task (Q4)

We evaluate the performance gain through our pre-training with the simplified task. We compare the BLEU score of the model with and without the pre-training while identically setting the other conditions. Table 9 shows that the student model results in a BLEU score reduction of 0.58 without pre-training, while achieving a lower BLEU score reduction of 0.27 with pre-training. Specifically, pre-training with a warm-up task reduces BLEU score by 0.31, which accounts for 53.44% of the total BLEU score reduction. The result indicates that the student model adapts well to the original challenging task after being pre-trained with the simplified task.

**Table 9:** Ablation study regarding pre-training with the simplified task. BLEU Reduction denotes the reduction in BLEU score from the uncompressed model’s score of 34.63. The accuracy of the model increases for PET when we apply the pre-training with the simplified task.

Condition	BLEU	BLEU Reduction
PET with the pre-training (proposed)	34.36	0.27
PET without the pre-training	34.05	0.58

### Effectiveness of mapping of prediction probabilities to labels in simplified task (Q5)

We compare our pre-training strategy with two naïve approaches to address the challenge due to the reduced number of classes in the simplified task in Table 10. The simplified task only has 4 classes while the original translation task has more than 3K classes. The two approaches address the problem by modifying the structure of the model: either reducing the dimension of the output layer or using an additional projection layer. Note that PET shows the highest BLEU score, accurately transferring the knowledge learned during pre-training to the training phase.

**Table 10:** Comparison of the BLEU scores according to different methods for mapping prediction probabilities to labels. PET maps prediction probabilities to labels of the simplified task, and achieves the best BLUE score. The proposed mapping approach makes the student model fully leverage the pre-trained parameters in KD, leading to improved performance.

Model	Method	BLEU
PET (proposed)	Map prediction probabilities to labels	34.36
Naïve approaches	Reduce the dimension of the output layer	30.89
	Use an additional projection layer	33.97

### Sensitivity analysis (Q6)

**Fig 6:**
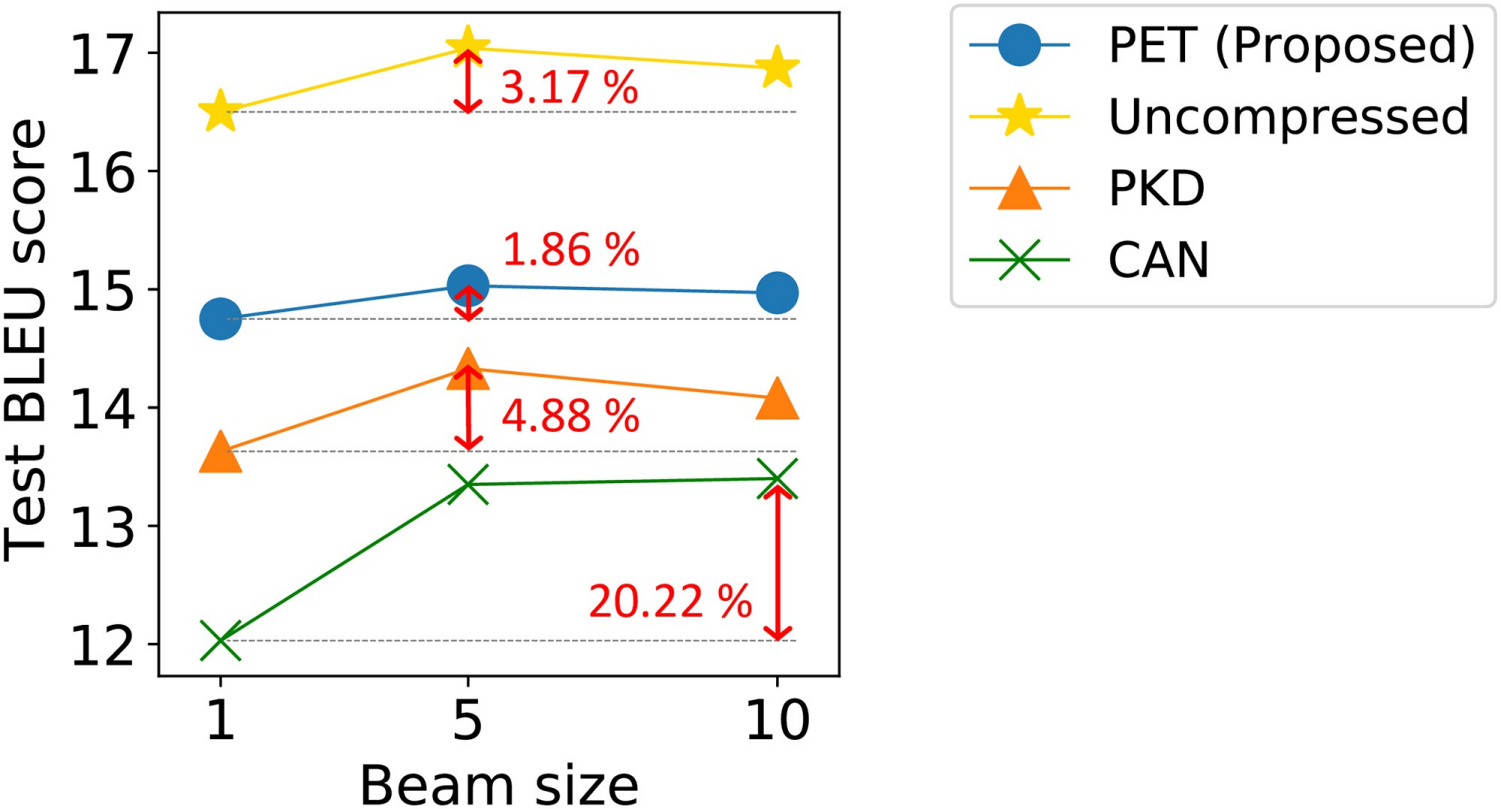
The translation accuracy with regard to the beam size. PET shows slight changes in the BLEU score, while the other competitors show significant fluctuations in the accuracy.

**The effect of the beam size**. We compare the BLEU score of PET and its competitors with respect to the beam size. In Fig 6, we show the result on the WMT’17 EN→LV task with the largest vocabulary and model size; results on other tasks are similar. Note that PET consistently shows the highest BLEU score among competitors over the various beam sizes. The difference between the highest and the lowest BLEU score of PET is 5.49× smaller than that of the second best method PKD. Also, PET shows a more robust result than the uncompressed model.

#### The effect of the random trials

We compare the BLEU score of PET and its competitors across random trials in English to German translation task. We first put validation and test data in one bucket and randomly divide the bucket into subset-L, M, and S. The three subsets consist of different numbers of sentences: 2992, 2048, and 1692, respectively. We perform translations with each subset and compare the number of translated tokens and the accuracy of the models. Table 11 shows the results. PET achieves the best translation accuracy among competitors. CAN translates more tokens but achieves lower accuracy than PET. This indicates that the number of translated tokens is not necessarily related to the translation accuracy. PET translates better by generating less number of tokens but in more accurate order.

**Table 11:** Comparison of the number of the translated tokens and BLEU scores in different sizes of data subsets. Subset-L, M, and S denote three randomly divided subsets of data with different sizes. PET consistently shows the best performance in all data subsets of varying sizes.

	Subset-L		Subset-M		Subset-S	
	#tokens	BLEU	#tokens	BLEU	#tokens	BLEU
Uncompressed	69,394	30.69	49,452	25.19	36,863	28.71
Patient-KD	68,495	27.61	48,838	22.92	36,316	25.75
CAN	68,892	27.52	49,406	22.90	36,675	25.42
**PET (proposed)**	**68,607**	**28.26**	**49,080**	**23.25**	**36,437**	**25.84**

### Limitations of PET

In PET, we focus on compressing the encoder and decoder of the Transformer, especially the attention modules. For the embedding and feed-forward layers, we simply select a reduced number of dimensions without utilizing advanced compression techniques when constructing the student model. Furthermore, the present version of PET distills only the output logits. We will study how to compress embedding and feed-forward layers of PET, and apply other recent KD approaches such as distillation from attention maps to obtain a more compact but accurate model for future work.

## Conclusion

We propose PET, an efficient Transformer compression method reducing the sizes of both the encoder and decoder. Two main ideas of PET are finding replaceable pairs for robust weight-sharing and designing a simplified task for pre-training. PET succeeds in achieving the memory efficiency and speed gain by up to 81.20% and 45.20%, respectively, with a small accuracy drop under 1%p. Future works include extending PET for extremely large language models, including GPT-3.
